# Supplementary material for: A novel integrative approach elucidates fine-scale dispersal patchiness in marine populations
Source: Sci Rep. 2019 Jul 25;9:10796. doi: 10.1038/s41598-019-47200-w (PMC6658486; doi:10.1038/s41598-019-47200-w)
Supplement: Supplementary file 1 — Supplementary Figures & Table [file 41598_2019_47200_MOESM1_ESM.docx]

**A novel integrative approach elucidates fine-scale dispersal patchiness in marine populations**

**C. Schunter^1^**^*^**, M. Pascual^2^, N. Raventos**^3^**, J. Garriga**^3^**, J.C. Garza^4^, F. Bartumeus**^3,5,6^, **E. Macpherson**^3^

^1^Swire Institute of Marine Science & School of Biological Sciences, University of Hong Kong, Pokfulam, Hong Kong SAR.

^2^Dept. Genètica, Microbiologia i Estadística - IRBio, Universitat Barcelona, Diagonal 643, 08028 Barcelona, Spain.

^3^Centre d’Estudis Avançats de Blanes (CEAB-CSIC), Car. Acc. Cala St. Francesc 14, Blanes, 17300 Girona, Spain.

**^4^**Southwest Fisheries Science Center, National Marine Fisheries Service and University of California, 110 McAllister Way, Santa Cruz 95060, USA.

**^5^**CREAF, E08193 Bellaterra (Cerdanyola del Vallès), Catalonia, Spain.

**^6^**ICREA, Passeig de Lluís Companys, 23, 08010 Barcelona, Spain.

SUPPLEMENTARY FIGURES & TABLE

Figure S1:

**Figure S1**: Correlation between body size and age according to otoliths reading for the 200 randomly selected juveniles.

Figure S2:

a)

b)

**Figure S2***:* Correlation between the date of juvenile hatching pelagic and a) larval duration (PLD) and b) settlement size established by otolith readings for the 200 randomly selected juveniles. a) Sea temperature at 3 meters depth increases across time during the studied area. The significance of the correlation was Pearson’s r= -0.6057, p<0.001.

Figure S3:


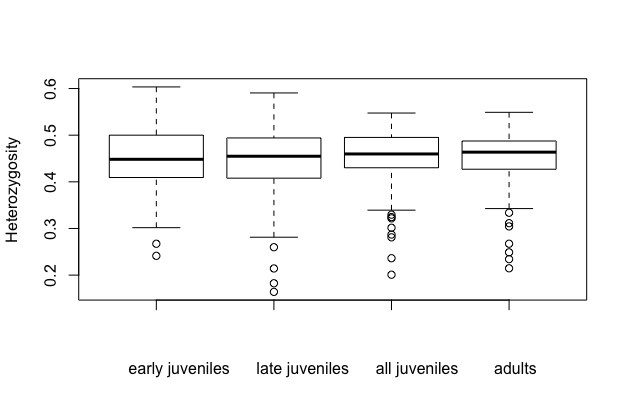


**Figure S3**: Heterozygosity boxplots for different life stage groupings. No difference in heterzygosity shows that there is no evidence of any sweep stake event.

Figure S4:

**
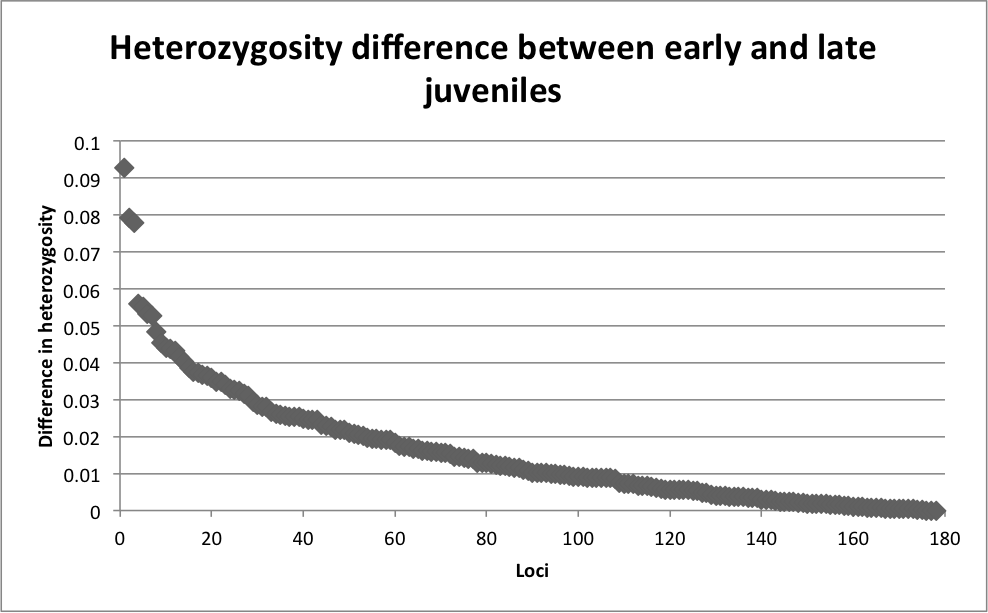
**

**Figure S4**: Absolute differences in heterozygosity between early and late juveniles for each of the 178 loci ordered from more to less differentiated.

Figure S5:

**Figure S5**: Box plots of relatedness values among different groups of individuals. Pairwise relatedness values do not decline across generations nor change within one recruitment season.

Figure S6:

a)


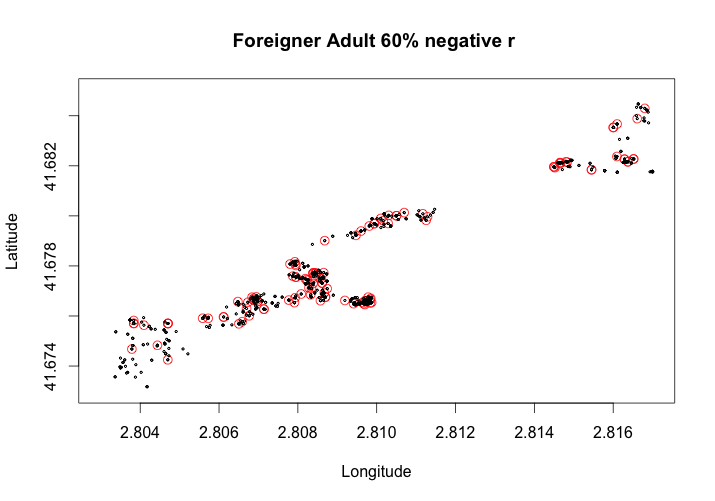


b)


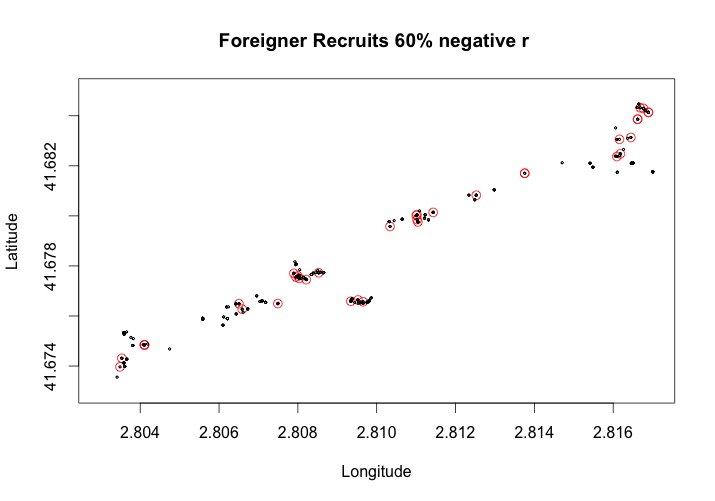


**Figure S6**: Spatial distribution of all individuals (black dots) and ‘recent migrant’ individuals (red dots) for adults (a) and juveniles (b) in the the small-scale area. ‘Recent migrants’ are the 25% of individuals (out of all adults or juveniles respectively) with the lowest ratios (bottom 25%). For adults it can be seen that there are less ‘recent migrants’ present in the southwestern part of the small sampling area.

Figure S7:


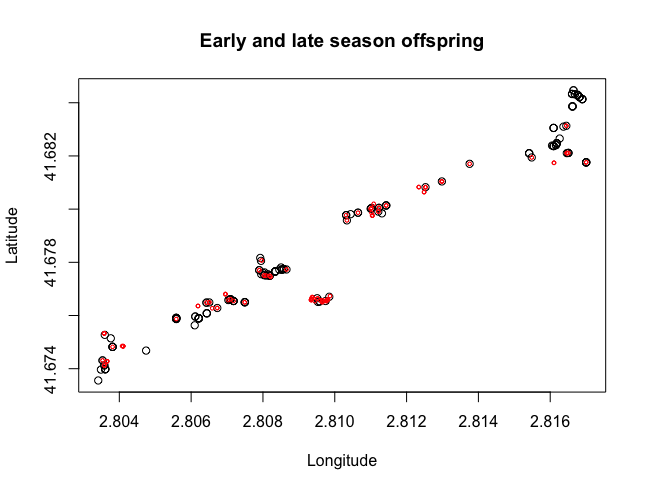


**Figure S7**: Spatial distribution of juveniles from the beginning of the season (black dots) and from the end of the season (red dots) in the small-scale area.

Figure S8:


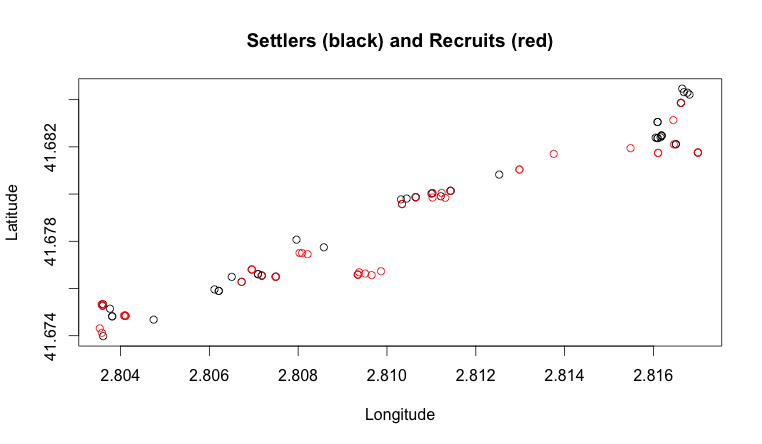


**Figure S8**: Spatial distribution of recent settlers (red dots) and survivors (black) to test for possible selective mortality or movement.

Table 1: Proportion of individuals in the small-scale area that belong to each of the four groups identified based on the combination of genetic differentiation within and between clusters (MIXED-range dispersers, SHORT-range dispersers, MEDIUM-range dispersers and LONG-range dispersers) for each spatial cluster displayed in Figure 3

| **Adults** | SOUTH |  |  |  |  |  | NORTH |
| --- | --- | --- | --- | --- | --- | --- | --- |
|  | SplClust1 | SplClust2 | SplClust3 | SplClust4 | SplClust5 |  | SplClust7 |
| N | 174 | 112 | 126 | 41 | 386 |  | 107 |
| MIXED-range dispersers | 0 | 0.06 | 0 | 0 | 0 |  | 0 |
| SHORT-range dispersers | **0.64** | **0.55** | **0.57** | **0.5** | 0.47 |  | 0.39 |
| MEDIUM range dispersers | 0.33 | 0.37 | 0.43 | 0.41 | **0.53** |  | **0.54** |
| LONG range dispersers | 0.04 | 0.02 | 0 | 0.09 | 0 |  | 0.07 |
| **Juveniles** | SplClust1 | SplClust2 | SplClust3 | SplClust4 | SplClust5 | SplClust6 | SplClust7 |
| N | 50 | 59 | 69 | 46 | 85 | 16 | 57 |
| MIXED-range dispersers | 0 | 0 | 0.2 | 0 | 0.26 | 0.5 | 0.12 |
| SHORT-range dispersers | 0.21 | 0.29 | 0.34 | 0.46 | **0.52** | 0.31 | **0.81** |
| MEDIUM-range dispersers | **0.79** | **0.71** | 0.36 | **0.54** | 0.12 | 0 | 0.07 |
| LONG-range dispersers | 0 | 0 | 0.1 | 0 | 0.1 | 0.19 | 0 |
| **Early juveniles** | SplClust1 | SplClust2 | SplClust3 | SplClust4 | SplClust5 | SplClust6 | SplClust7 |
| N | 11 | 28 | 27 | 29 | 13 | 2 | 20 |
| MIXED-range dispersers | 0 | 0.11 | 0.29 | 0 | 0 | 0.26 | 0.15 |
| SHORT-range dispersers | 0.29 | 0.13 | 0.43 | 0 | **0.63** | 0.47 | 0.41 |
| MEDIUM-range dispersers | 0.41 | **0.53** | 0.29 | 0 | 0.38 | 0.26 | 0.36 |
| LONG-range dispersers | 0.29 | 0.24 | 0 | 1 | 0 | 0 | 0.08 |
| **Late juveniles** | SplClust1 | SplClust2 | SplClust3 | SplClust4 | SplClust5 | SplClust6 | SplClust7 |
| N | 12 | 33 | 17 | 20 | 18 | 10 | 11 |
| MIXED-range dispersers | 0.30 | 0.41 | 0.3 | **0.58** | 0.28 | 0.5 | 0 |
| SHORT-range dispersers | 0.45 | 0 | 0 | 0.18 | 0.11 | 0 | **0.64** |
| MEDIUM-range dispersers | 0.25 | 0 | 0.4 | 0.12 | 0 | 0 | 0.36 |
| LONG-range dispersers | 0 | **0.59** | 0.3 | 0.12 | 0.61 | 0.5 | 0 |

Clustering for adults only, juveniles only, early juveniles (those that settled early, before mid-May, during the reproductive period) and late juveniles (those that settled late during the reproductive period, after mid-June) is shown. SplClust = Spatial cluster corresponding to the clusters in Figure 3. Note that SplClust6 is not present in adults. MIXED-range dispersers: individuals with high mean relatedness (r) with respect to individuals in their own spatial cluster (rIN) and high r with respect to individuals in other spatial clusters (rOUT). SHORT-range dispersers: individuals with high rIN and low rOUT. MEDIUM-range dispersers: individuals with low rIN and high rOUT. LONG-range dispersers: individuals with low rIN and low rOUT. Values above 0.5 are in bold.
